# Supplementary material for: Induction of Superior Systemic and Mucosal Protective Immunity to SARS-CoV-2 by Nasal Administration of a VSV–ΔG–Spike Vaccine
Source: Vaccines (Basel). 2024 May 1;12(5):491. doi: 10.3390/vaccines12050491 (PMC11125831; doi:10.3390/vaccines12050491)
Supplement: Supplementary file 1 [file vaccines-12-00491-s001.zip › vaccines-2949550-supplementary.pdf]

Supp. Table 1:

a

IgG - Tukey's multiple comparisons test	
**10^3 IN+IN vs. 10^3 IM+IM**	***
10^3 IN+IN vs. 10^4 IM+IM	**
**10^3 IN+IN vs. 10^3 IM+IN**	**
10^3 IN+IN vs. 10^4 IM+IN	*
10^4 IN+IN vs. 10^3 IM+IM	****
**10^4 IN+IN vs. 10^4 IM+IM**	****
10^4 IN+IN vs. 10^6 IM+IM	**
10^4 IN+IN vs. 10^3 IM+IN	****
**10^4 IN+IN vs. 10^4 IM+IN**	***
10^4 IN+IN vs. 10^5 IM+IN	*
10^4 IN+IN vs. 10^6 IM+IN	*
10^5 IN+IN vs. 10^3 IM+IM	****
10^5 IN+IN vs. 10^4 IM+IM	***
10^5 IN+IN vs. 10^3 IM+IN	***
10^5 IN+IN vs. 10^4 IM+IN	**

b

IgA - Tukey's multiple comparisons test	
10^3 IN+IN vs. 10^5 IN+IN	**
10^4 IN+IN vs. 10^3 IM+IM	**
**10^4 IN+IN vs. 10^4 IM+IM**	*
10^5 IN+IN vs. 10^3 IM+IM	****
10^5 IN+IN vs. 10^4 IM+IM	**
**10^5 IN+IN vs. 10^5 IM+IM**	*
10^5 IN+IN vs. 10^3 IM+IN	**
10^5 IN+IN vs. 10^4 IM+IN	**
**10^5 IN+IN vs. 10^5 IM+IN**	**

Supplementary table S1: Statistical significance of humoral immunity following mucosal or muscular vaccination: (a) Statistical analysis of induction of IgG by IM+IM vaccination, IM+IN vaccination, or IN+IN vaccination, at various doses. (b) Statistical analysis of IgA by IM+IM vaccination, IM+IN vaccination, or IN+IN vaccination, at various doses. Analysis was performed using One-way ANOVA, with Tukey's Post Hoc test, *$p < 0.05$; ** $p < 0.01$; *** $p < 0.001$; **** $p < 0.0001$. Matched doses are in Bold.

Supp. Fig. S1:

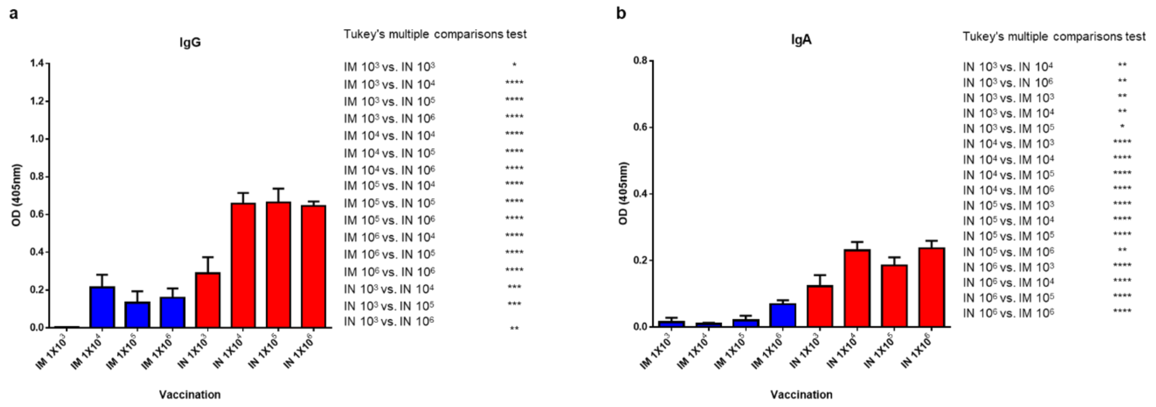

Supplementary Figure S1: Induction of humoral immunity following a prime only mucosal or muscular vaccination: (a) Induction of IgG by IM or IN vaccination at various doses. Each color represents route of administration. (b) Induction of IgA by IM or IN vaccination at various doses. Each color represents route of administration. Data is presented as mean ± SEM. n=8 for each group. Statistical analysis for each panel is presented. Analysis was performed using One-way ANOVA, with Tukey's Post Hoc test, *$p < 0.05$; ** $p < 0.01$; *** $p < 0.001$; **** $p < 0.0001$.

Supp. Fig. S2:

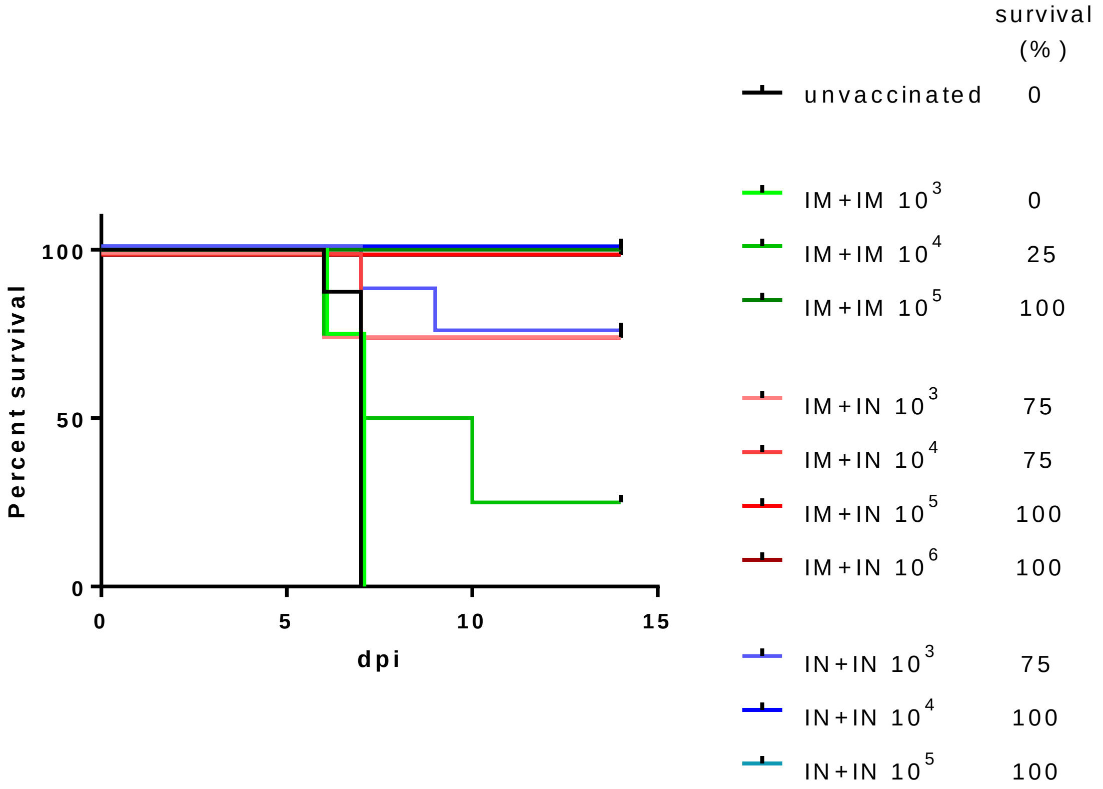

Supplementary Figure S2: Protection of mice from lethal SARS-CoV-2 challenge by mucosal or muscular vaccination: Survival curve of K18-hACE2 mice vaccinated by IM+IM, IM+IN, or IN+IN regimens at doses ranging between 10^3-10^6 pfu, or unvaccinated, challenged by IN instillation with Delta variant of SARS-CoV-2. Statistical analysis was performed using One-way ANOVA, with Tukey's Post Hoc test. Significance is indicated next to figure legend. n=8 for unvaccinated and IN+IN vaccinated groups, n=4 for IM+IM and IM+IN groups.

Supp. Fig. S3:

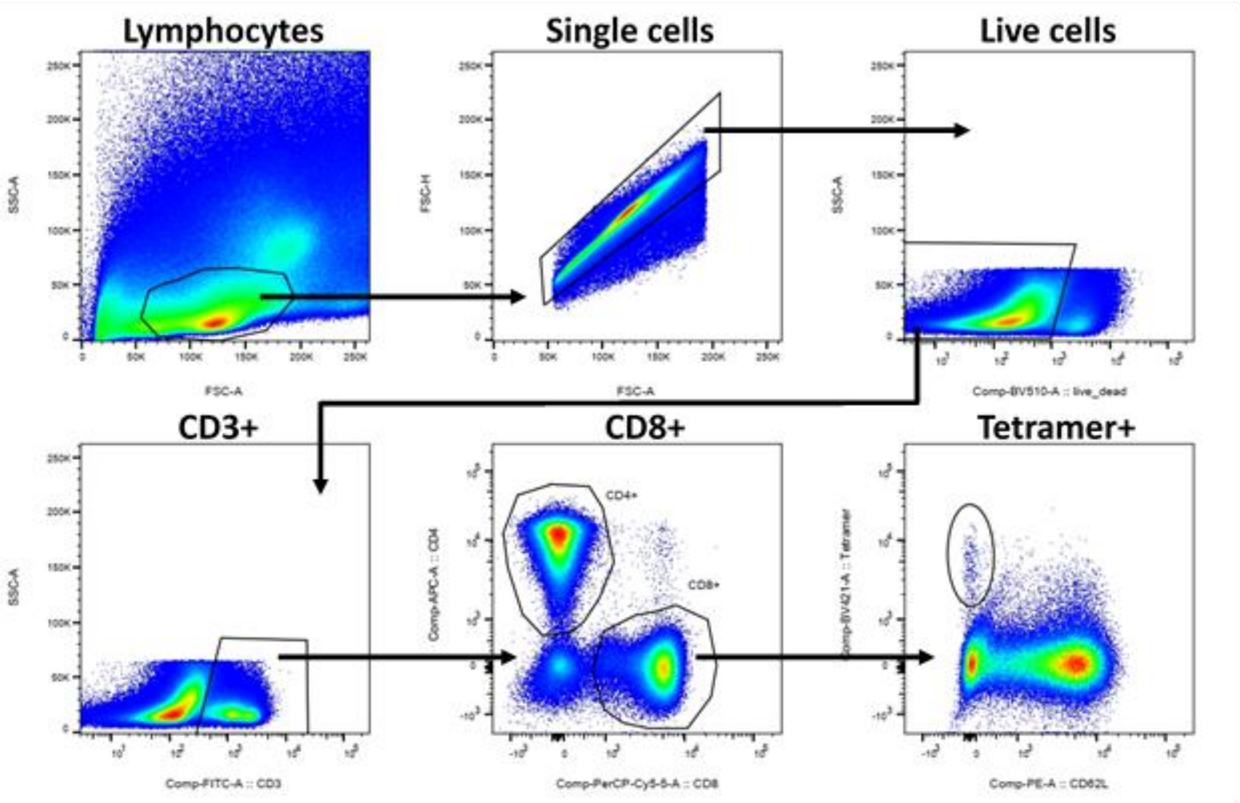

Supplementary Figure S3: Gating strategy for evaluation of effector, spike specific CD8+ T-cells in the Lung.

Supp. Fig. S4:

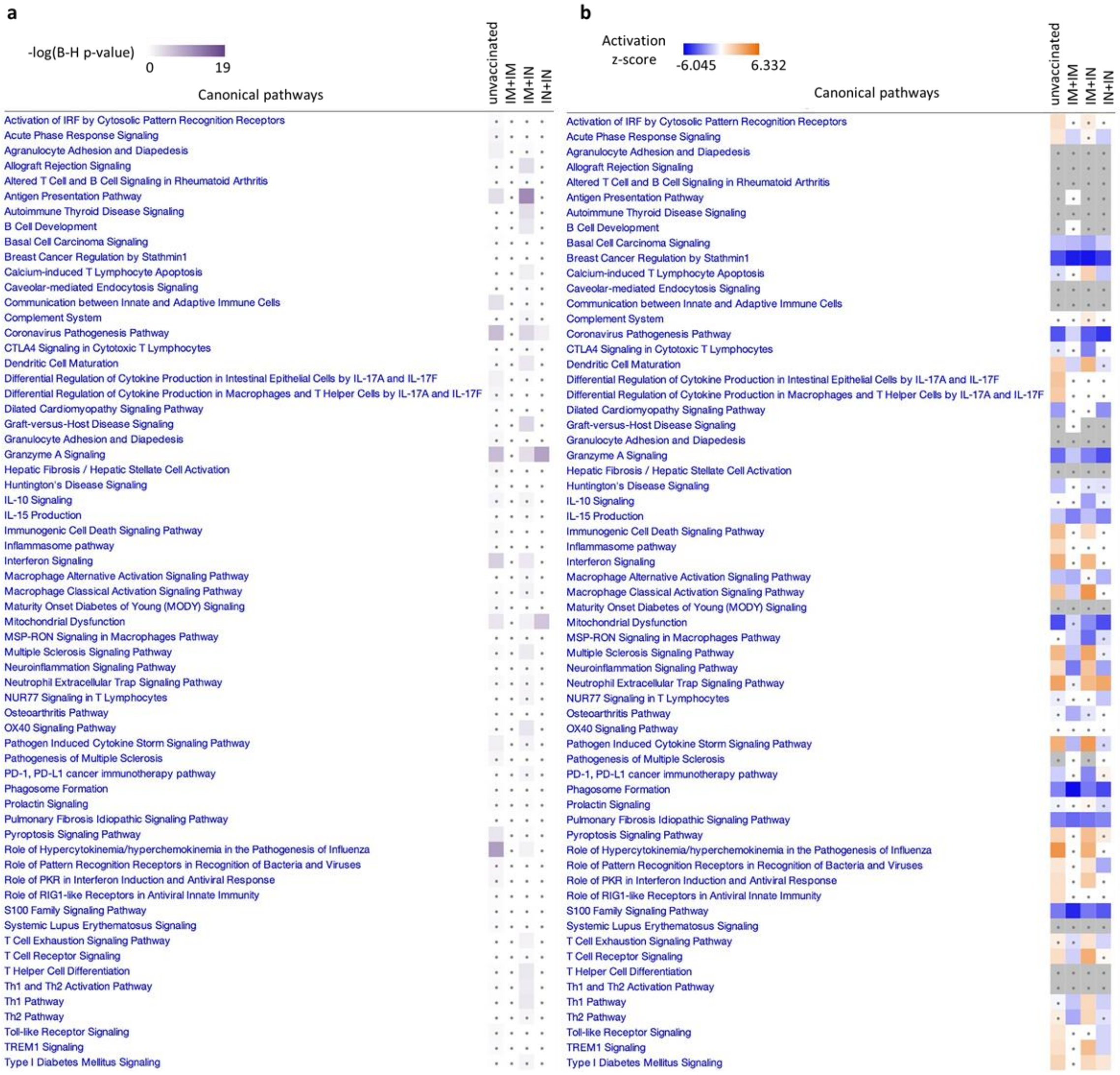

Supplementary Figure S4: Pathway comparison analysis of unvaccinated and vaccinated mice brains: (a-b) Ingenuity Pathway Analysis (IPA) enrichment analysis in comparison of unvaccinated and infected mice brains, compared to IM+IM, IM+IN, or IN+IN vaccinated and infected mice brains. (a) Predicted enriched canonical pathways, arranged by -log(p-value), (b) and activation z-score. Orange indicates predicted activation, blue indicates predicted inhibition of the pathway. Darker colors indicate higher absolute (a) -log(B-H p-value) or (b) z-scores. Pathways are arranged in an alphabetical order. n=3 for each group. Right-tailed Fisher's Exact test followed by Benjamini-Hochberg multiple comparisons correction test were performed.

Supp. Fig. S5:

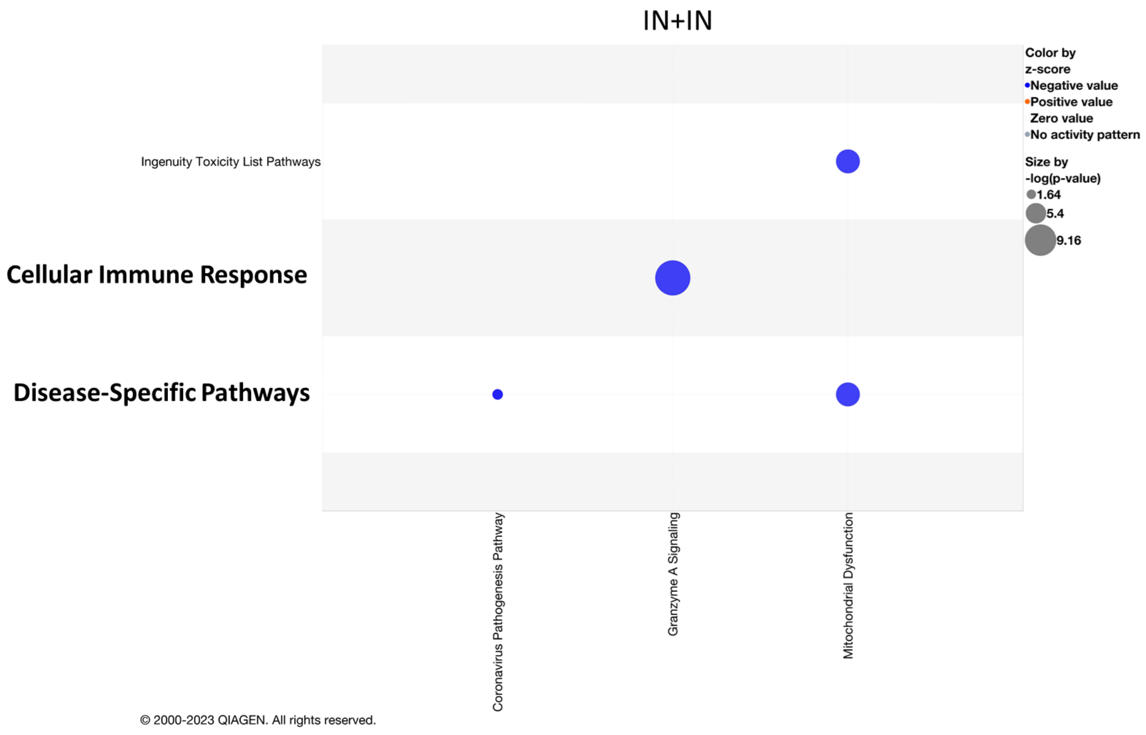

Supplementary Figure S5: Reduced enrichment of cellular and humoral immune responses, cytokine signaling and disease and pathogen influenced signaling by homologous IN vaccination: Ingenuity pathway analysis bubble plot for IN+IN vaccinated K18-hACE2 mice brains at 4 days post delta variant SARS-CoV-2 infection. Enriched pathways arranged by categories of interest: cellular immune response, cytokine signaling, disease-specific pathways, humoral immune response and pathogen-influenced signaling (y-axis), and the enriched canonical pathways that map to each category (x-axis), arranged in an alphabetical order. Blue predicts decreased activation, bubble size represents -log(p-value). IPA was set to a cutoff of -log (p-value) of 1.3 (threshold), and adjusted p-value <0.05. n=3. The significance of the association between the data set and specific canonical pathways was accessed using a right-tailed Fisher's Exact test. A Benjamini-Hochberg multiple comparisons correction test was applied.
